# Prognostication of serial post-intensity-modulated radiation therapy undetectable plasma EBV DNA for nasopharyngeal carcinoma

**DOI:** 10.18632/oncotarget.14137

**Published:** 2016-12-24

**Authors:** Victor Ho-Fun Lee, Dora Lai-Wan Kwong, To-Wai Leung, Cheuk-Wai Choi, Vincent Lai, Lydia Ng, Ka-On Lam, Sherry Chor-Yi Ng, Chun-Kin Sze, Chi-Chung Tong, Patty Pui-Ying Ho, Wing-Lok Chan, Lai-San Wong, Dennis Kwok-Chuen Leung, Sum-Yin Chan, Pek-Lan Khong

**Affiliations:** ^1^ Department of Clinical Oncology, Queen Mary Hospital, Li Ka Shing Faculty of Medicine, The University of Hong Kong, Hong Kong; ^2^ Department of Diagnostic Radiology, Li Ka Shing Faculty of Medicine, The University of Hong Kong, Hong Kong; ^3^ Department of Radiation Oncology, University of Southern California, Los Angeles, California, USA

**Keywords:** intensity-modulated radiation therapy, nasopharyngeal carcinoma, non-metastatic, plasma EBV DNA, prognostic factors

## Abstract

Plasma Epstein-Barr virus (EBV) DNA titers have been used to monitor treatment response and provide prognostic information on survival for nasopharyngeal carcinoma (NPC). However, the long-term prognostic role of pretreatment and posttreatment titers after radical contemporaneous radiation therapy remains uncertain. We recruited 260 evaluable patients with non-metastatic NPC treated with radical intensity-modulated radiation therapy (IMRT) with or without adjunct chemotherapy. Plasma EBV DNA titers at baseline and then 8 weeks and 6 months after IMRT were measured. Cox regression models were employed to identify interaction between post-IMRT 8^th^ week and 6^th^ month undetectable titers and 3-year survival endpoints. Concordance indices (Ct) from time-dependent receiver-operating characteristics (TDROC) were compared between patients with post-IMRT undetectable and those with detectable titers. After a median follow-up duration of 3.4 years (range 1.4-4.6 years), patients with post-IMRT 8^th^ week and 6^th^ month undetectable plasma EBV DNA titers enjoyed longer 3-year survival endpoints than those who had detectable titers at the same time points. Post-IMRT 8^th^ week, and more significantly, post-IMRT 6^th^ month undetectable plasma EBV DNA were the only significant prognostic factors of 3-year survival endpoints. Ct values for all 3-year survival endpoints for both post-IMRT 8^th^ week and 6^th^ month undetectable plasma EBV DNA were significantly higher in those with stage IVA–IVB diseases compared to stage I-III counterparts. Early post-IMRT undetectable plasma EBV DNA titers were prognostic of 3-year survival endpoints in patients with non-metastatic NPC. Intensified treatment should be further explored for patients with persistently detectable titers after IMRT.

## INTRODUCTION

Nasopharyngeal carcinoma (NPC) is unique in its geographical distribution with prevalence in Southern China, Hong Kong, Taiwan, Singapore and Malaysia as well as association with Epstein-Barr virus (EBV) [1, 2]. According to global cancer registry, NPC ranked 11^th^ most common among all malignancies in China in 2008 with an incidence of 2.8/100000 person-years in men and 1.9/100000 person-years in females. The most common histological types are undifferentiated non-keratinizing carcinoma (WHO Type III) and differentiated non-keratinizing carcinoma (WHO Type II). Radiation therapy alone is offered for stage I and II disease while concurrent chemoradiation is indicated for locally advanced stage III to IVB disease [3]. Intensity-modulated radiation therapy (IMRT) has been the most contemporaneous radiation therapy technique for the past decade due to its superior tumor coverage and dose sparing of critical normal structures from unnecessary radiation, leading to better treatment outcomes and toxicity profiles [4–11]. Plasma EBV DNA titer has been found important in both diagnosis and monitoring of treatment response during and after definitive treatment [12–22]. However, its role in the IMRT era remains uncertain, and the appropriate time points for its surveillance after IMRT are undefined. Currently plasma EBV DNA titer has yet to be recommended by the American Joint Committee on Cancer (AJCC) staging manual and European Society for Medical Oncology (ESMO) as a tumor marker in routine clinical staging and posttreatment monitoring [23]. There is an unmet and urgent need for identifying its role at baseline and in posttreatment monitoring to improve risk stratification, leading to closer surveillance and intensified treatment in poor-risk groups in the modern era of radiation therapy. We initiated this prospective observational study on measuring baseline and serial plasma EBV DNA titers for all patients with newly diagnosed non-metastatic NPC to determine the prognostic value of early post-IMRT plasma EBV DNA on long-term survival outcomes. We now presented our preliminary results on 3-year survival endpoints. This study was registered with the National Cancer Trial Registry as NCT02476669 (ClinicalTrials.gov).

## RESULTS

**Figure 1:**
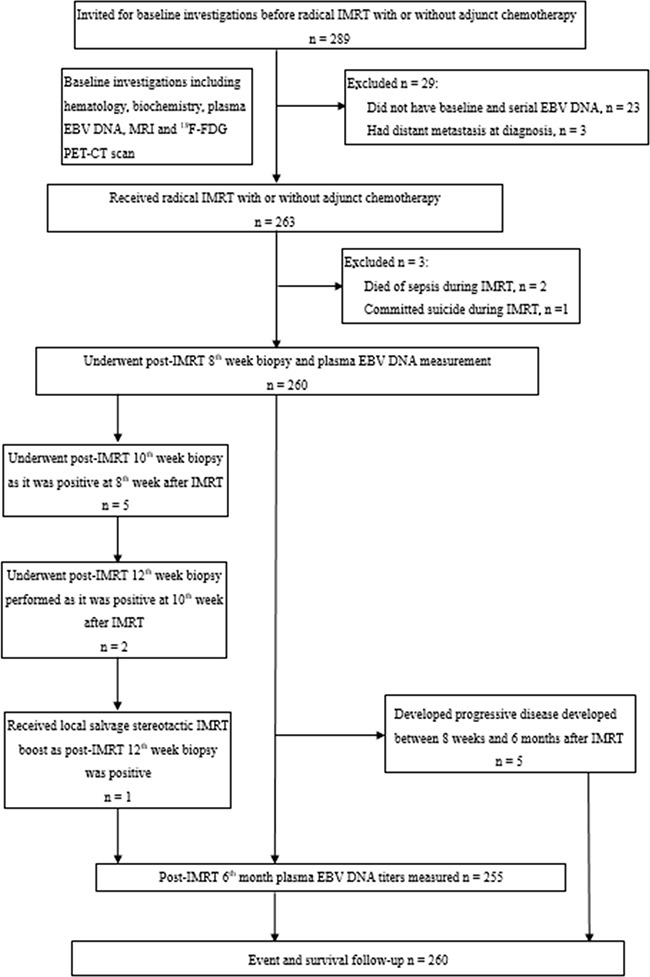
Participant flow in the current study DNA: deoxyribonucleic acid, EBV: Epstein-Barr virus, 18F-FDG: [18F]fluorodeoxyglucose, IMRT: intensity-modulated radiation therapy, MRI: magnetic resonance imaging, NPC: nasopharyngeal carcinoma, PET-CT: positron-emission tomography with integrated contrast-enhanced computed tomography.

The study schematic diagram was shown (Figure 1). A total of 289 consecutive patients were recruited from July 2010 to June 2013. Of them, 29 patients were excluded from subsequent analyses after written informed consent: 23 patients did not have blood taken for baseline or serial plasma EBV DNA, three patients suffered from stage IVC metastatic disease confirmed by PET-CT scan and another three patients died during IMRT (two died from sepsis and one committed suicide). The characteristics of the remaining 260 patients for analysis were displayed in Table 1.

**Table 1:** Characteristics of study patients

Characteristic	n = 260
	No. of patients (%)
Mean age in years (range)	53 (16–91)
Male/female	183 (70.4)/77 (29.6)
ECOG performance status	
0	16 (6.2)
1	244 (93.8)
T-classification	
T1	77 (29.6)
T2	28 (10.8)
T3	115 (44.2)
T4	40 (15.4)
N-classification	
N0	30 (11.5)
N1	74 (28.5)
N2	121 (46.5)
N3a	4 (1.5)
N3b	31 (11.9)
Overall stage	
I	17 (6.5)
II	38 (14.6)
III	135 (51.9)
IVA	35 (13.5)
IVB	35 (13.5)
Laterality of primary tumor	
Midline	142 (54.6)
Left	57 (21.9)
Right	61 (23.5)
Retropharyngeal node present	200 (76.9)
Laterality of retropharyngeal node (n = 200)	
Ipsilateral	88 (44.0)
Contralateral	3 (1.5)
Bilateral	109 (54.5)
Median baseline plasma EBV DNA in copies/ml (range)	934 (10–1575000)
Stage I (n = 17)	13 (10–210)
Stage II (n = 38)	746 (10–21250)
Stage III (n = 135)	847 (11–175000)
Stage IVA (n = 35)	1400 (10–90500)
Stage IVB (n = 35)	7625 (12–1575000)
Radical IMRT only	46 (17.7)
Concurrent chemoradiation	26 (10.0)
Induction chemotherapy then concurrent chemoradiation	89 (34.2)
Concurrent chemoradiation then adjuvant chemotherapy	99 (38.1)

DNA: deoxyribonucleic acid, EBV: Epstein-Barr virus, ECOG: Eastern Cooperative Oncology Group, IMRT: intensity-modulated radiation therapy.

### Treatment outcomes after IMRT and their plasma EBV DNA at different time points

**Figure 2:**
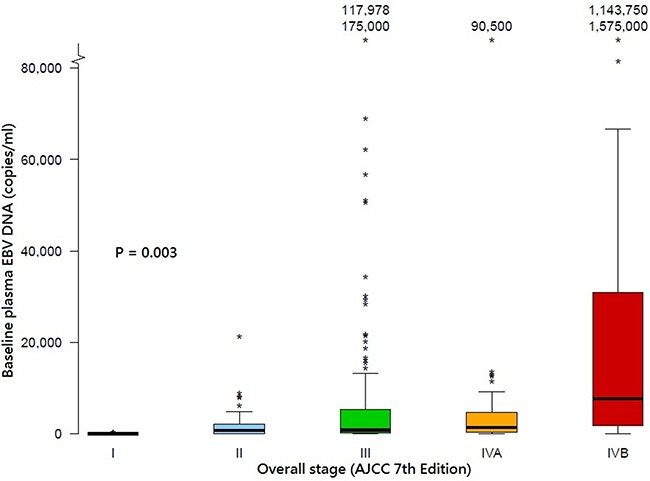
Box and whisker plot showing the distribution of baseline plasma EBV DNA titers across different overall stages of NPC in the study population The band inside the boxes indicates the median. The boxes indicate the interquartile range and the whiskers indicate the values between (1st quartile - step) and (3rd quartile + step) where step = 1.5 × interquartile range. The * indicates an extreme outlier with its value either 2 steps below the 1st quartile or 2 steps above the 3rd quartile. AJCC: American Joint Committee on Cancer; DNA, deoxyribonucleic acid, EBV: Epstein-Barr virus.

The median baseline plasma EBV DNA titers for all patients was 934 copies/ml (range 10–1575000 copies/ml). Mood's median test showed that the medians were significantly different across different overall stages (*P* = 0.003) (Figure 2). All patients completed IMRT without treatment interruption. Forty-six patients received IMRT alone for their early-stage disease (40 patients) or because of their advanced age and/or significant medical contraindications to chemotherapy (6 patients). However, these 6 patients were still free from relapse. At 8 weeks after IMRT, 30 patients still had elevated plasma EBV DNA (range 14–4512 copies/ml); all but 1 had undetectable titers at 12 weeks after IMRT and this remaining patient's plasma EBV DNA was then undetectable at 16 weeks after IMRT. Another patient, despite undetectable plasma EBV DNA, had confirmed local persistence at 12 weeks. He received additional stereotactic IMRT boost (20Gy in 8 fractions over 1.5 weeks) without any residual disease noted afterwards. His plasma EBV DNA was persistently undetectable before and after stereotactic boost. Five patients developed progressive disease with distant metastases between 8 weeks and 6 months after IMRT, and thus they were only included in the subsequent TDROC and survival analysis using post-IMRT 8^th^ week (but not 6^th^ month) undetectable plasma EBV DNA as stratifying factor. Seventeen patients had elevated plasma EBV DNA (range 19–29375 copies/ml) at 6 months after IMRT. Of them, 3 developed local recurrence, 1 patient suffered from regional nodal failure, 5 patients had distant metastases, 1 patient had both regional and distant failure, 1 patient had local and distant failure and the remaining 6 were still free of relapse. After a median follow-up duration of 3.4 years (range 1.4–4.6 years), 10, 5 and 19 patients had local failure, regional failures and distant metastases, translated into 96.2%, 98.1% and 92.7% for the 3-year local, regional and distant metastasis control rates respectively. The 3-year LFFS, RFFS, DMFS, PFS, CSS and OS were 95.2%, 96.9%, 91.2%, 88.0%, 95.8%, 93.9% respectively. The Kaplan-Meier estimates of all survival endpoints for all stages were also shown (Supplementary Figure S1).

### Correlation of post-IMRT 8^th^ week and 6^th^ month undetectable plasma EBV DNA with long-term survival endpoints

**Figure 3:**
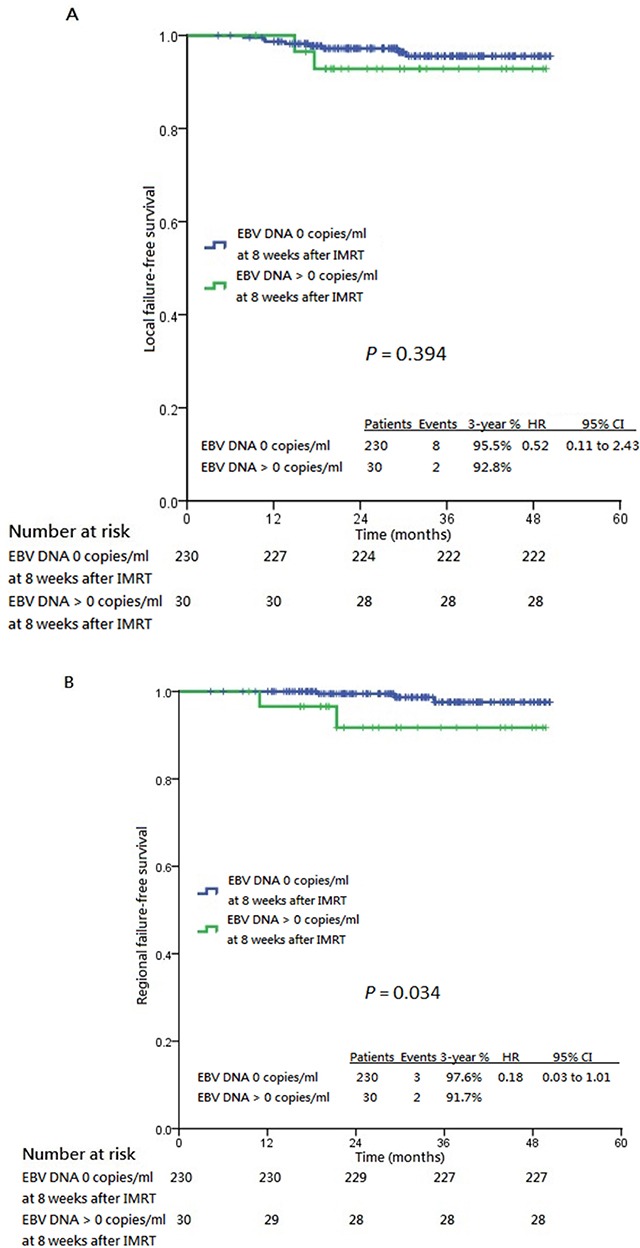

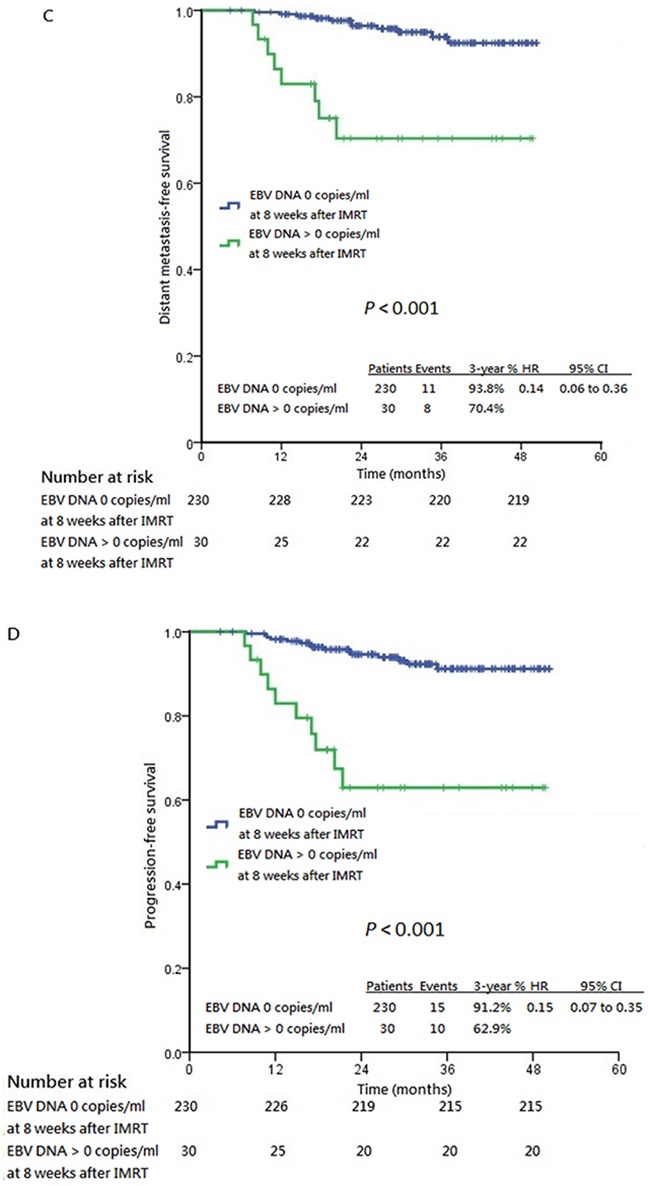

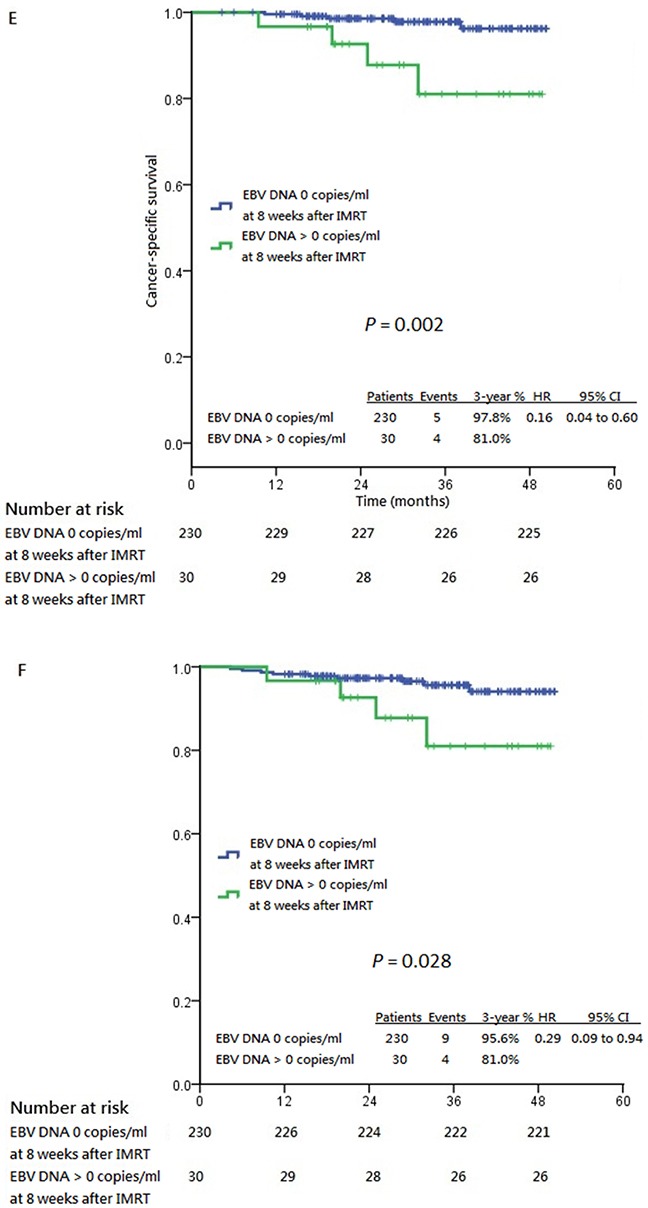
Kaplan-Meier estimates of all survival endpoints stratified by post-IMRT 8th week undetectable plasma EBV DNA Panel **A**. shows local failure-free survival. Panel **B**. shows regional failure-free survival. Panel **C**. shows distant metastasis-free survival. Panel **D**. shows progression-free survival. Panel **E**. shows cancer-specific survival and Panel **F**. shows overall survival, all stratified by post-IMRT 8th week plasma EBV DNA 0 copies/ml and post-IMRT 8th week plasma EBV DNA >0 copies/ml. CI: confidence interval, HR: hazard ratio, IMRT: intensity-modulated radiation therapy.

**Figure 4:**
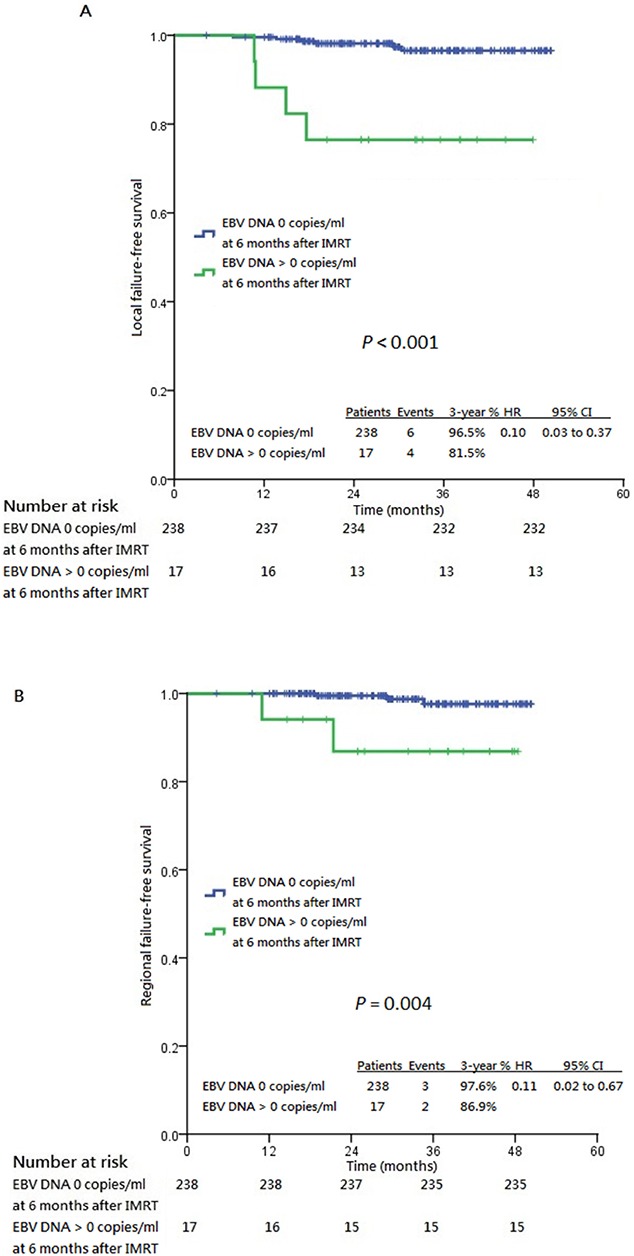

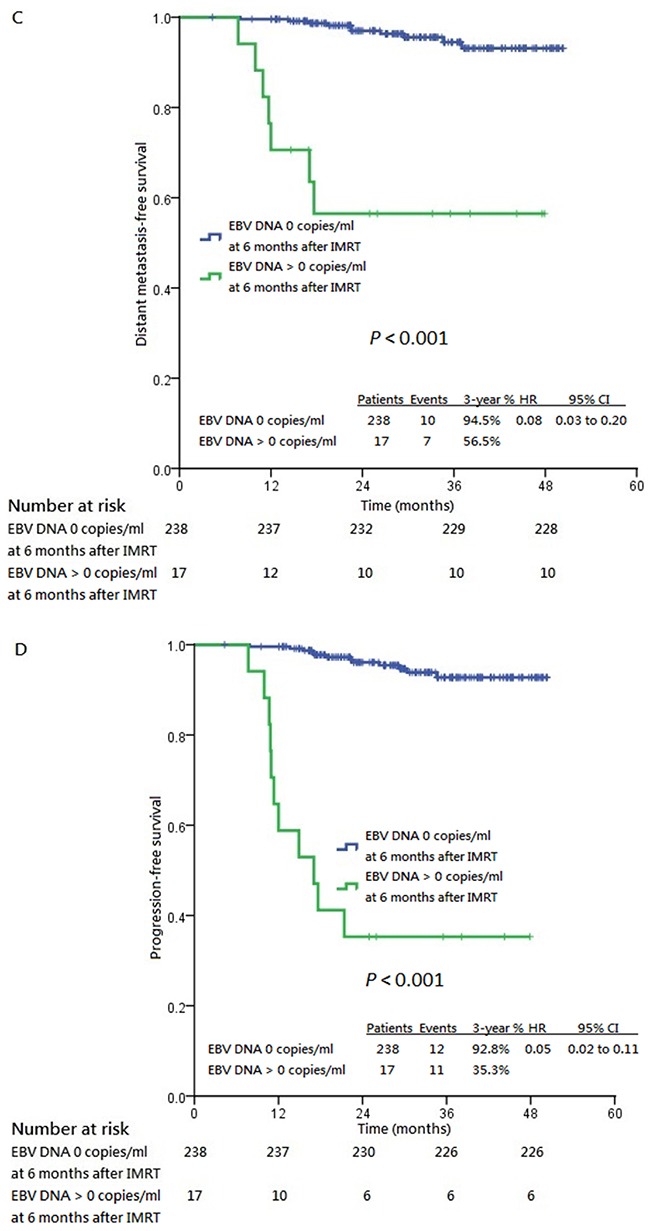

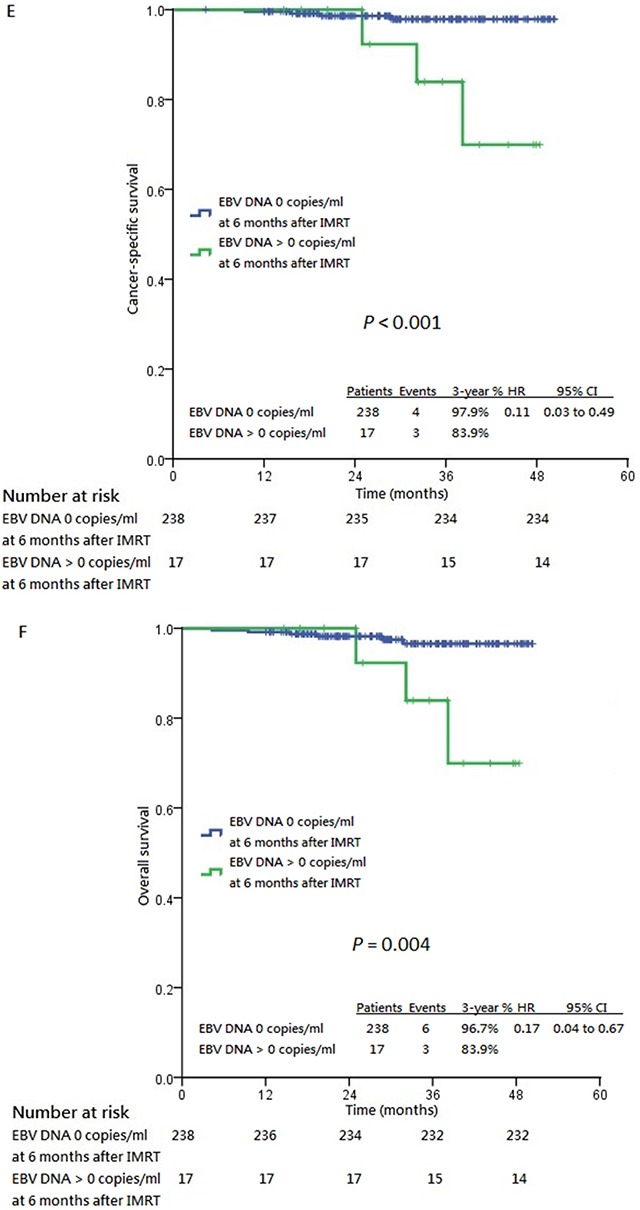
Kaplan-Meier estimates of all survival endpoints stratified by post-IMRT 6th month undetectable plasma EBV DNA Panel **A**. shows local failure-free survival. Panel **B**. shows regional failure-free survival. Panel **C**. shows distant metastasis-free survival. Panel **D**. shows progression-free survival. Panel **E**. shows cancer-specific survival and Panel **F**. shows overall survival, all stratified by post-IMRT 6th month plasma EBV DNA 0 copies/ml and post-IMRT 6th month plasma EBV DNA >0 copies/ml. CI: confidence interval, HR: hazard ratio, IMRT: intensity-modulated radiation therapy.

Patients who had post-IMRT 8^th^ week and 6^th^ month undetectable plasma EBV DNA enjoyed longer 3-year survival endpoints compared to those who had post-IMRT detectable titers at the corresponding time points, except LFFS stratified by post-IMRT 8^th^ week undetectable plasma EBV DNA (Table 2, Figure 3 and Figure 4). In particular, greater survival differences were revealed between patients with undetectable and detectable post-IMRT 6^th^ month plasma EBV DNA. The prognostic significances between post-IMRT 8^th^ week and 6^th^ month undetectable plasma EBV DNA and the survival endpoints were further evaluated in univariable and multivariable Cox models (Supplementary Table S1 to Table S3). Only post-IMRT 8^th^ week and 6^th^ month undetectable plasma EBV DNA were prognostic of all survival endpoints (except for LFFS stratified by post-IMRT 8^th^ week undetectable plasma EBV DNA), while baseline plasma EBV DNA was prognostic of LFFS, RFFS, DMFS and PFS only (Supplementary Table S1 to Table S3).

**Table 2:** Impact of undetectable plasma EBV DNA at 8 weeks and 6 months after IMRT on various survival endpoints

	Post-IMRT 8th week plasma EBV DNA			Plasma IMRT 6^th^ month plasma EBV DNA		
	0 copies/ml	> 0 copies/ml	*P*	0 copies/ml	> 0 copies/ml	*P*
LFFS			0.394			< 0.001
Median (months)	NR	NR		NR	NR	
Mean (months)	49.0	47.3		49.4	39.8	
95% CI	48.1–49.9	44.2–50.5		48.6–50.2	32.8–46.8	
HR	0.52			0.10		
95% CI	0.11 to 2.43			0.03-0.37		
RFFS			0.034			0.004
Median (months)	NR	NR		NR	NR	
Mean (months)	49.9	47.0		49.9	44.3	
95% CI	49.3–50.4	43.4–50.6		49.3–50.4	38.9–49.7	
HR	0.18			0.11		
95% CI	0.03–0.98			0.02-0.67		
DMFS			< 0.001			< 0.001
Median (months)	NR	NR		NR	NR	
Mean (months)	48.5	39.0		48.7	32.6	
95% CI	47.4–49.6	32.7–45.3		47.7–49.7	23.9–41.2	
HR	0.14			0.08		
95% CI	0.06–0.36			0.03–0.20		
PFS			< 0.001			< 0.001
Median (months)	NR	NR		NR	NR	
Mean (months)	47.8	36.7		48.4	25.4	
95% CI	46.6–49.1	30.2–43.2		47.4–49.5	17.4–33.4	
HR	0.16			0.05		
95% CI	0.07–0.35			0.02–0.11		
CSS			0.002			< 0.001
Median (months)	NR	NR		NR	NR	
Mean (months)	49.5	44.8		49.7	43.8	
95% CI	48.8–50.3	40.4–49.2		49.1–50.3	39.3–48.4	
HR	0.16			0.11		
95% CI	0.04–0.60			0.03-0.49		
OS			0.028			0.004
Median (months)	NR	NR		NR	NR	
Mean (months)	48.8	44.8		49.4	43.8	
95% CI	47.8–49.8	40.4–49.2		48.6–50.2	39.3–48.4	
HR	0.29			0.17		
95% CI	0.09–0.94			0.04–0.67		

CI: confidence interval, CSS: cancer-specific survival, DMFS: distant metastasis-free survival, HR: hazard ratio, IMRT: intensity-modulated radiation therapy, LFFS: local failure-free survival, NR: not reached, OS: overall survival, PFS: progression-free survival, RFFS: regional failure-free survival.

TDROC analysis revealed significantly higher Ct indices and AUC values in stage IVA to IVB diseases compared to stage I to III counterparts for all survival endpoints using both post-IMRT 8^th^ week and 6^th^ month undetectable plasma EBV DNA as stratification (Supplementary Table S4 and Supplementary Figure S2). In addition, the Ct indices and AUC values were consistently higher for post-IMRT 6^th^ month undetectable plasma EBV DNA compared to post-IMRT 8^th^ week undetectable plasma EBV DNA. These indicated that post-IMRT undetectable plasma EBV DNA possessed a higher predictive power of survival endpoints for more advanced stage disease than earlier stage disease and the power was stronger with post-IMRT 6^th^ month undetectable plasma EBV DNA than with post-IMRT 8^th^ week undetectable titers. This was also illustrated in ordinary ROC analyses with, in general, higher AUC values for post-IMRT 6^th^ month undetectable plasma EBV DNA in each survival endpoint (Supplementary Figure S3).

## DISCUSSION

The discovery of plasma EBV DNA has established itself as an accurate biomarker for detection, surveillance and prognostication of NPC. For the past 15 years, numerous studies have investigated the correlation between pretreatment plasma EBV DNA levels and clinical stage, tumor load and treatment outcomes [12–21]. However, either these studies were performed when the 5^th^ and 6^th^ edition of AJCC staging system were in use, when modern radiation therapy techniques for example IMRT was not fully implemented or when various cut-off values of pretreatment and posttreatment plasma EBV DNA titers were used in the exploratory analyses. In addition, PET-CT scan as complete and accurate staging workup was not employed in previous studies; thus, some patients might have been understaged. Though Peng et al in their recent retrospective study showed the prognostic value of post-IMRT plasma EBV DNA on survival outcomes, blood taking for plasma EBV DNA was checked at only one time point after IMRT and the exact date of measurement was not specified [24]. On the contrary, our study is the first prospective observational studies investigating the prognosticative role of baseline and serial post-IMRT plasma EBV DNA titers on various predefined survival endpoints on the basis of complete PET-CT staging and full IMRT implementation.

In addition, the method of measuring plasma EBV DNA was in line with that mentioned in the previous study be Le et al which correlated the plasma EBV DNA titers of 40 samples in each of the four tertiary referral centers in the United States, Taiwan and Hong Kong [25]. The EBV DNA copy numbers were all determined by real-time quantitative polymerase chain reaction (qPCR) (BamHI-Wprimer/ probe). A large variabiltiy of plasma EBV DNA titers was observed, especially when different PCR master mixes and calibrators were used. After study harmonization and standardization with the use of same master mixes and calibrators, correlations were more than 99% between the centers. A uniform and consistent method of EBV DNA assay should be employed in future multi-center prospective studies.

Our excellent treatment results were likely attributed to complete radiological staging, dedicated IMRT contouring and planning, and close posttreatment surveillance. The median survival was thus not reached in all subgroups due to a lack of failure events. This also explained why the other prespecified baseline covariates including stage and baseline plasma EBV DNA were not good segregators of survival difference. Our preliminary 3-year survival endpoints are also representative indicators of long-term survival outcomes, as only 0 (0%), 0 (0%) and one (0.4%) patient developed local, regional and distant failure respectively at more than three years after IMRT. This echoed the large retrospective study in China which included 868 patients with non-metastatic NPC treated with IMRT with or without adjunct chemotherapy, with the vast majority of treatment failures occurring within the 1^st^ to 3^rd^ year after treatment [26]. This highlighted the crucial importance of frequent surveillance within the first three years after IMRT. Of course longer follow up with more mature results is needed in our study for the detection of late relapses.

Ordinary ROC, as a tool to evaluate the accuracy of biomarkers with a contemporaneous reference standard, is not robust to predict future events especially survival outcomes at a future time point. Instead, TDROC employs another reference standard which refers to an event at a future time and may not be known for every patient due to early censoring [27]. This is particularly favorable to predict survival of a highly curable cancer with few failure and survival events [28]. We found that, from TDROC analysis, higher Ct indices and AUC values were observed in stage IVA to IVB diseases compared to stage I to III counterparts for all survival endpoints using both post-IMRT 8^th^ week and 6^th^ month undetectable plasma EBV DNA as stratification. In addition, the Ct indices and AUC values were consistently higher for post-IMRT 6^th^ month undetectable plasma EBV DNA compared to post-IMRT 8^th^ week undetectable plasma EBV DNA. It could be inferred that post-IMRT undetectable plasma EBV DNA possessed a higher predictive power of survival endpoints for more advanced stage disease than earlier stage disease and the power was stronger with post-IMRT 6^th^ month than with post-IMRT 8^th^ week undetectable titers. We speculated that those with more advanced stage disease had higher baseline plasma EBV DNA titers and so post-IMRT undetectable EBV DNA titers should better reflect a higher chance of complete disease remission. Besides, 5 (1.9%) patients developed progressive disease between 8^th^ week and 6^th^ month after IMRT and thus only 255 patients were included in the subsequent time-dependent ROC (TDROC) and survival analysis at 6 months after IMRT. This might be one possible reason why post-IMRT 6^th^ month undetectable plasma EBV DNA was more predictive of survival endpoints compared to post-IMRT 8^th^ week undetectable plasma EBV DNA titers.

Our study demonstrated that most treatment failures were distant metastases following superior locoregional control by IMRT. It may be inferred from our study that it is probably not late for patients to consider additional intensified treatment at 8 weeks after IMRT if their plasma EBV DNA is still detectable, provided that undetectable plasma EBV DNA could be achieved at 6 months after IMRT. This begs the question as to whether additional or more intensified chemotherapy for patients with persistently elevated post-IMRT plasma EBV DNA titers can reduce distant relapse and improve survival. Twu et al demonstrated that adjuvant oral tegafur-uracil for one year with or without preceding intravenous chemotherapy improved distant relapse and OS in patients with persistently detectable plasma EBV DNA taken one week after completion of radiation therapy [29]. This has so far been the only published study indicating a survival benefit of adjuvant chemotherapy in those with persistently elevated plasma EBV DNA after completion of RT. Recently in the largest multi-institutional prospective study in Hong Kong, Hui et al demonstrated that posttreatment, instead of pretreatment, plasma EBV DNA titers were prognostic of relapse-free survival and OS [30]. However, the optimal timing of checking posttreatment plasma EBV DNA has yet to be defined since it may take longer time for complete clearance of plasma EBV DNA after radiation therapy as reported in a previous study [31]. Our current study showed that 30 patients (11.5%) still had persistently elevated plasma EBV DNA at eight weeks after IMRT, but 20 patients had complete plasma EBV DNA clearance at later time points without evidence of relapse. Moreover, IMRT was not fully implemented in the two aforementioned studies. Whether additional or more intensified adjuvant chemotherapy brings survival benefit may be deciphered by the ongoing Hong Kong NPC-0502 study (NCT00370890), which is designed to assess the benefit of adjuvant chemotherapy in patients at high risk of disease recurrence, identified with detectable plasma EBV DNA at 6 weeks after chemoradiation [32]. The combined phase II and III NRG-HN001 trial (ClinicalTrials.gov NCT02135042) investigating the role of different adjuvant chemotherapy regimens (standard cisplatin and 5-FU regimen versus gemcitabine and paclitaxel combination) as a phase II setting and omission of adjuvant chemotherapy in those with undetectable plasma EBV DNA following IMRT may certainly offer a clearer answer to this unresolved question [33]. However, this will pose another clinical question on the equipoise between survival prolongation and chemotherapy-related toxicities by the administration of additional adjuvant chemotherapy. Chen et al in their phase III multicenter randomized-controlled trial concluded that adjuvant chemotherapy following concurrent chemoradiation failed to improve survival as compared concurrent chemoradiation alone [34]. Of note, 82% in the concurrent chemoradiation plus adjuvant chemotherapy group started adjuvant chemotherapy and only 63% completed all cycles of adjuvant chemotherapy. Patient refusal was the principal reason for uncompleted adjuvant chemotherapy. In addition, 69% and 49% of patients who received adjuvant chemotherapy had treatment delays and dose reduction respectively, because of adverse events and other reasons. The infrequent use of IMRT in less than half of the study population was another study limitation. Despite lack of improvement in all prespecified survival endpoints, numerically the results favored adjuvant chemotherapy with a 2% absolute difference in failure-free survival (*P* = 0.13), distant failure-free survival (*P* = 0.12) and OS. Since this study was not designed as a non-inferiority trial against concurrent chemoradiation alone, we cannot strictly conclude that adjuvant chemotherapy brings no benefits in locally advanced NPC. Induction chemotherapy before concurrent chemoradiation, on the other hand, may improve treatment compliance and possibly better systemic control. However, conflicting results were reported in different prospective studies [35, 36]. More recently, Lee et al in the preliminary analysis of their NPC-0501 study revealed that induction chemotherapy with cisplatin and capecitabine (PX) reflected a significant reduction in the hazards of disease progression and possibly death compared to induction chemotherapy with cisplatin and 5-FU (PF) and adjuvant PF regimen [37]. As a whole, our results suggested that it is inadequate to apply only the TNM staging system for participant selection in clinical trials, and that use of biomarkers will probably enhance the power of future clinical trials to obtain positive results. Our study provided a new insight into the change in the current international practice guidelines of non-metastatic NPC treated with IMRT. Personalized treatment intensification should be considered for those who show early serological signs of relapse, which should be confirmed in prospective randomized-controlled trials.

In conclusion, early serial undetectable plasma EBV DNA titers after radical IMRT with or without adjunct chemotherapy were prognostic of better 3-year survival outcomes in patients with non-metastatic nasopharyngeal carcinoma.

## MATERIALS AND METHODS

### Study design and population

Patients with newly diagnosed non-metastatic NPC of undifferentiated type (WHO Type III) treated with IMRT as a curative treatment were recruited into this prospective observational study since July 2010 with prior approval by the local institutional review board of our institution. The study was undertaken in accordance with Declaration of Helsinki and Good Clinical Practice guidelines. After written informed consent, all patients had pretreatment workup including serum hematology and biochemistry, plasma EBV DNA, [18F]fluorodeoxyglucose (^18^F-FDG) positron-emission tomography with integrated contrast-enhanced computed tomography (PET-CT) scan with patients immobilized by a prefabricated customized head and neck thermoplastic cast, and magnetic resonance imaging (MRI) including T1, T2 and fat-suppressed contrast-enhanced T1 sequences of the head and neck in treatment position by 3T scanner (Arhieva 3.0 T, Philips Healthcare, Best, The Netherlands) on the following day as previously described to exclude those with distant metastasis or second primary malignancy and for IMRT target delineation and planning (Supplementary Data). Two independent radiologists specialized in head and neck radiology and blinded to treatment details determined the stage and tumor extent; any discrepancies were resolved by consensus, based on the 7^th^ edition of AJCC classification. Plasma EBV DNA was extracted and calculated as previously described (Supplementary Data) [12]. All patients also underwent endoscopy and nasopharyngeal biopsies on both sides of the nasopharynx to assess extent of mucosal tumor spread. Then they received IMRT as described (Supplementary Data) [38]. Briefly, 70 Gy was delivered to the planning target volume (PTV)-70 and 66 Gy to the PTV-66 in 33 fractions by the simultaneous accelerated radiation therapy technique. In general, patients with stage I and II disease received IMRT alone while stage III to IVB patients received concurrent chemoradiation with the Intergroup 0099 regimen consisting of cisplatin intravenous infusion (100mg/m^2^) every three weeks starting on the 1^st^ day of IMRT and adjuvant chemotherapy with cisplatin on day 1 and 5-FU from day 1 to 4 for three more cycles starting at four weeks following IMRT [39]. Patients with bulky cervical nodal (≥3cm in diameter) stage II disease were also given concurrent chemoradiation only, at the discretion of the treating oncologist. Patients who had their primary tumors in close proximity to normal critical organs-at-risk (OARs) would receive 3 cycles of induction chemotherapy (cisplatin 100mg/m^2^ on day 1 and 5-FU 1000mg/m^2^ from day 1 to 5 every three weeks) before concurrent chemoradiation, in an attempt for significant tumor shrinkage so that a radical radiation dose could be delivered to the tumors with more sparing of these OARs. At 8 weeks following completion of IMRT, all patients had routine 6-site random nasopharyngeal biopsies as our standard practice and blood checked for plasma EBV DNA titers (Figure 1) [40]. Local salvage treatment was given to patients who had persistent primary tumor at 12 weeks after IMRT [41]. If their plasma EBV DNA was still >0 copies/ml at 8 weeks after IMRT, it would be repeated 4 weeks thereafter until it was undetectable or it was proven to have persistent locoregional disease or metastasis clinically or radiologically. Patients with complete local remission had follow up clinical evaluation every 2 to 3 months, surveillance MRI scan every 3 to 4 months, and PET-CT scan if clinically suspicious of relapse. Plasma EBV DNA was measured again at 6 months after IMRT and then as clinically indicated afterwards. Any elevation of plasma EBV DNA would lead to endoscopy and nasopharyngeal biopsy, MRI and PET-CT evaluation and subsequent salvage treatment if recurrence was confirmed.

### Statistical analysis

The prespecified survival endpoints including local failure-free survival (LFFS), regional failure-free survival (RFFS), distant metastasis-free survival (DMFS), progression-free survival (PFS), cancer-specific survival (CSS) and overall survival (OS) were defined and evaluated by log rank tests (Supplementary Data). Univariable and multivariable analyses by Cox proportional hazard models were employed for identifying prognostic factors for these survival endpoints with age, sex, Eastern Cooperative Oncology Group (ECOG) performance status (PS), T-classification, N-classification, overall stage of NPC, IMRT alone versus chemoradiation, concurrent chemoradiation only, induction chemotherapy then concurrent chemoradiation, concurrent chemoradiation then adjuvant chemotherapy and baseline and post-IMRT 8^th^ week and 6^th^ month undetectable plasma EBV DNA titers as covariates. Time-dependent receiver-operating characteristics (TDROC) analyses were performed to obtain time-dependent concordance indices (Ct) and area under the curve (AUC) for advanced stage IVA to IVB disease versus stage I to III disease [42]. Nonparametric bootstrapping with 1000 replicates was also used to determine 95% confidence interval (CI) of the Ct and to evaluate the difference in Ct indices between these disease groups. TDROC was performed by the statistical software package R version 3.1.3 [43]. The other statistical analyses were performed by Statistical Package for Social Sciences (SPSS) version 20. Tests were considered statistically significant at *P* < 0.05 (two-sided). The database-lock date for analysis was December 30, 2015.

This study was presented in part as an oral abstract at 51^st^ American Society of Clinical Oncology (ASCO) Annual Meeting (Abstract 6007), Chicago, IL, May 29-June 2, 2015. Clinical trial information: NCT02476669 (ClincalTrials.gov).

## SUPPLEMENTARY MATERIALS FIGURES AND TABLES










