# Supplementary material for: Prognostication of serial post-intensity-modulated radiation therapy undetectable plasma EBV DNA for nasopharyngeal carcinoma
Source: Oncotarget. 2016 Dec 24;8(3):5292–308. doi: 10.18632/oncotarget.14137 (PMC5354909; doi:10.18632/oncotarget.14137)
Supplement: Supplementary file 1 [file oncotarget-08-5292-s001.pdf]

Prognostication of serial post-intensity-modulated radiation therapy undetectable plasma EBV DNA for nasopharyngeal carcinoma

SUPPLEMENTARY MATERIALS AND METHODS

Determination of plasma EBV DNA titers

In brief, four milliliters (ml) of peripheral blood was drawn and placed in an EDTA tube. A total of about 400–800 microliters of plasma samples were used for DNA extraction by a QIAamp Blood Kit (Qiagen, Hilden, Germany). The exact amount of plasma was determined for calculation of EBV DNA genome copies. Circulating EBV DNA concentrations were measured using a real-time quantitative polymerase chain reaction (PCR) system with ABI Prism® 7000 Sequence Detection System (Applied Biosystems, USA) that amplified a DNA segment in the *Bam*HI-W fragment region of the EBV genome. All plasma DNA samples were also subject to real-time PCR analysis for the β-*globin* gene, which gave a positive signal on all tested samples. Multiple controls without templates were also included in each analysis as negative controls. All results were expressed as EBV DNA genome copies per ml with accuracy to the nearest 0.1 copies/ml. Undetectable plasma EBV DNA meant 0 copies/ml and they were used interchangeably in the main text and the Supplementary Data.

Imaging examinations for NPC staging at baseline

Two independent radiologists (Vincent Lai and Pek-Lan Khong) who were specialized in head and neck radiology and blinded to study treatment details reviewed all PET-CT and MRI images to determine the stage and tumor extent. Any discrepancies were resolved by consensus. All of the imaging scans were performed by the same PET-CT and MRI scanners at Department of Diagnostic Radiology, Queen Mary Hospital, The University of Hong Kong throughout the whole study period.

PET-CT scan protocol

Whole body [18F]fluorodeoxyglucose (^{18}F-FDG) PET-CT was performed with a combined PET-CT scanner (Discovery VCT, 64 multislice spiral CT; GE Healthcare Bio-Sciences Corp) using a standardized protocol. All patients were immobilized in a supine treatment position by a custom-made thermoplastic head and neck cast before scanning. The scanning range was from the vertex of brain to the upper thigh. After six hours of fasting, 220 to 370 MBq [4.8 MBq/kilogram (kg)] of body weight-adjusted ^{18}F-FDG was administered intravenously. After a 60-minute uptake time, whole-body emission PET scans were obtained with seven bed positions. Attenuation-corrected PET images with CT data were reconstructed with an ordered-subset expectation maximisation iterative reconstruction algorithm (14 subsets and two iterations) and fused with CT images (Advanced Workstation 4.3; GE Healthcare Bio-Sciences). The CT imaging parameters were as follows: 120kVp; 200 to 400 mA; 0.5 second per CT rotation; pitch 0.984:1; and 2.5 mm intervals, with or without 60 to 100 ml (1.5 ml/kg of body weight) intravenous contrast medium.

MRI techniques

MRI scanning was performed on the following day after PET-CT scanning with a 3.0-T MR scanner (Achieva 3.0T, Philips Healthcare, Best, The Netherlands), utilizing a 16-channel neurovascular coil. Image acquisition was made in axial plane from suprasellar region cranially to lung apices caudally. Four standard sequences were performed: a) axial T1-weighted turbo spin echo (TSE) [repetition time/echo time (TR/TE) = 454/9.2 milliseconds (ms); turbo factor = 3; FOV = 230 × 230 millimeter (mm); matrix = 672 × 672; slice number = 32; slice thickness = 3 mm; intersection gap = 0.3 mm]; b) axial T2-weighted short TI inversion recovery (STIR) [TR/TE = 4644/60 ms; field-of-view [FOV] = 230 × 230 mm; matrix = 672 × 672; slice number = 32; slice thickness = 3 mm; intersection gap = 0.3 mm]; c) coronal T2-weighted STIR [TR/TE = 4644/60 ms; FOV = 230 × 230 mm; matrix = 480 × 480; slice number = 32; slice thickness = 3 mm; intersection gap = 0.3 mm]; d) 3D T1-weighted turbo-field- echo (TFE) post-contrast scan [TR/TE = 4.8/2.4 ms; flip angle = 100; FOV = 230 × 230 mm; matrix = 640 × 640; slice number = 319; slice thickness = 0.72 mm]. Intravenous bolus injection of 0.1 mmol/kg of body weight gadopentetate dimeglumine was then administered at 1.5 ml per second for post-contrast acquisition.

IMRT planning protocol

Every patient was immobilized in the supine position during 18F-FDG PET-CT acquisition and actual treatment by using a thermoplastic head and neck cast. A customized mouthguard was fabricated for better immobilization. MRI images mentioned above were co-registered with the planning PET-CT images for dedicated delineation

of the target volumes and organs-at-risk (OARs). OARs, including brainstem, spinal cord, globes, optic nerves, optic chiasm, lenses, temporomandibular joints, temporal lobes, auditory nerves, cochleae, mandible, oral cavity, larynx, parotid glands and vestibules were first contoured. Then gross tumor volumes (GTV) of both the primary tumor and the radiologically involved cervical nodes were outlined. Subsequently, the clinical target volume (CTV-70) for the microscopic disease spread and planning target volume containing CTV-70 with a 5-mm margin (PTV-70) to take into account physiological body motion and set-up errors were generated respectively. Another CTV-66 encompassing the high-risk areas including the posterior half of the maxillary sinuses, nasal cavities, parapharyngeal spaces, styloid processes, basiocciput, basisphenoid, clivus, foramina rotunda and ovale, pterygopalatine fossae, pterygomaxillary fissures, infraorbital fissures, cavernous sinuses, and level Ib and V nodal stations were also outlined subsequently. A corresponding PTV-66 with a 3-mm margin encompassing the CTV-66 was created by Boolean operations of the treatment planning system (Eclipse version 8.0 to 10.0 software, Eclipse Treatment Planning System, Palo Alto, CA, USA), which was also used for IMRT planning using Analytical Anisotropic Algorithm. All the targets and OARs delineation were approved by senior radiation oncologists (Victor Lee, Dora Kwong and To-Wai Leung) before dose optimization for IMRT. During optimization, the maximum dose to the brainstem, optic nerves, and chiasm was limited to 54 Gy and less than 45 Gy to the spinal cord. Allowance was given for some locally advanced tumors in which the maximum dose to the brainstem, optic nerves and chiasm could be up to 60 Gy. Efforts were also made to limit the mean dose to the parotid glands to 26 Gy and the dose to the lenses and temporal lobes to as low as could reasonably be achieved without compromising dose coverage to the PTVs. A dose of 70 Gy was prescribed to the PTV-70 and 66 Gy to the PTV-66 in 33 fractions delivered by the simultaneous accelerated radiation therapy technique (SMART). If there was no clinical nodal disease in the patients' lower neck, either an extended IMRT field or a separate anterior field matched with a monoisocentric technique to the IMRT field above was employed based on the oncologist's preference and 66Gy in 33 fractions were prescribed for the anterior neck field if present. All IMRT planning, dose optimization and quality assurance was performed by a certified medical physicist (Sherry Ng) and all IMRT plans fulfilled acceptance criteria with at least 95% of PTVs having received the prescribed dose, the maximum dose of PTVs limited to 107% or below and the maximum dose of organs-at-risk within tolerance limits according to International Commission on Radiation Units and Measurements (ICRU) criteria. They were then approved by senior radiation oncologists (Victor Lee, Dora Kwong and To-Wai Leung) before IMRT commencement. Positional verification with on-board imaging was performed before and then daily before the first 3 fractions of IMRT followed by weekly afterwards during the whole course of IMRT, to track any anteroposterior and lateral body displacements.

Post-IMRT surveillance and follow up

Eight weeks following completion of IMRT, all patients underwent nasoendoscopy again with routine 6-site random nasopharyngeal biopsies at both roofs, lateral and posterior walls of the nasopharynx. If residual tumors were noted histologically, they needed another endoscopy and biopsies two weeks later as positive histological findings may undergo spontaneous remission with time, as we reported previously. Another endoscopy and biopsies would be performed again at 12th week after completion of IMRT if there was still residual tumor at 10th week after completion of IMRT. Local persistence, as we defined for more than 15 years in our institution, was tumor persistence in nasopharyngeal biopsies at 12 weeks after IMRT and patients would receive salvage treatment for instance intracavitary brachytherapy boost, stereotactic radiotherapy etc. If all 6-site biopsies were negative, patients were considered to have complete local remission. They also had plasma EBV DNA checked again on the same day of nasoendoscopy at eight weeks after IMRT completion. If their EBV DNA was still >0 copies/ml, it would be repeated every four weeks thereafter until it was undetectable or until it was proven to have persistent local or regional disease, or distant metastasis. For patients with complete local remission, they would have regular follow-up every two to three months for any relapse and complications, as well as MRI scan every three to four months and PET-CT scan if clinically suspicious of relapse. Plasma EBV DNA was also taken at six months after IMRT and then if clinically indicated afterwards for those who had undetectable (i.e. 0 copies/ml) plasma EBV DNA eight weeks after IMRT.

Survival endpoints

Prespecified survival endpoints in this study include local failure-free survival (LFFS), regional failure-free survival (RFFS), distant metastasis-free survival (DMFS), progression-free survival (PFS), cancer-specific survival (CSS) and overall survival (OS). LFFS was defined as the time from the date of diagnosis of NPC to the date of local recurrence or death from any cause. RFFS was defined as the time from date of diagnosis of NPC to the date of regional neck recurrence or death from any cause.

DMFS was defined as the time from date of diagnosis to the date of distant metastasis or death from any cause. PFS was defined as the time from date of diagnosis of NPC to the date of any form of disease progression or death from any cause. CSS were defined as the time from date of diagnosis of NPC to the date of cancer-related death. OS was defined as the time from the date of diagnosis of NPC to the date of death from any cause.

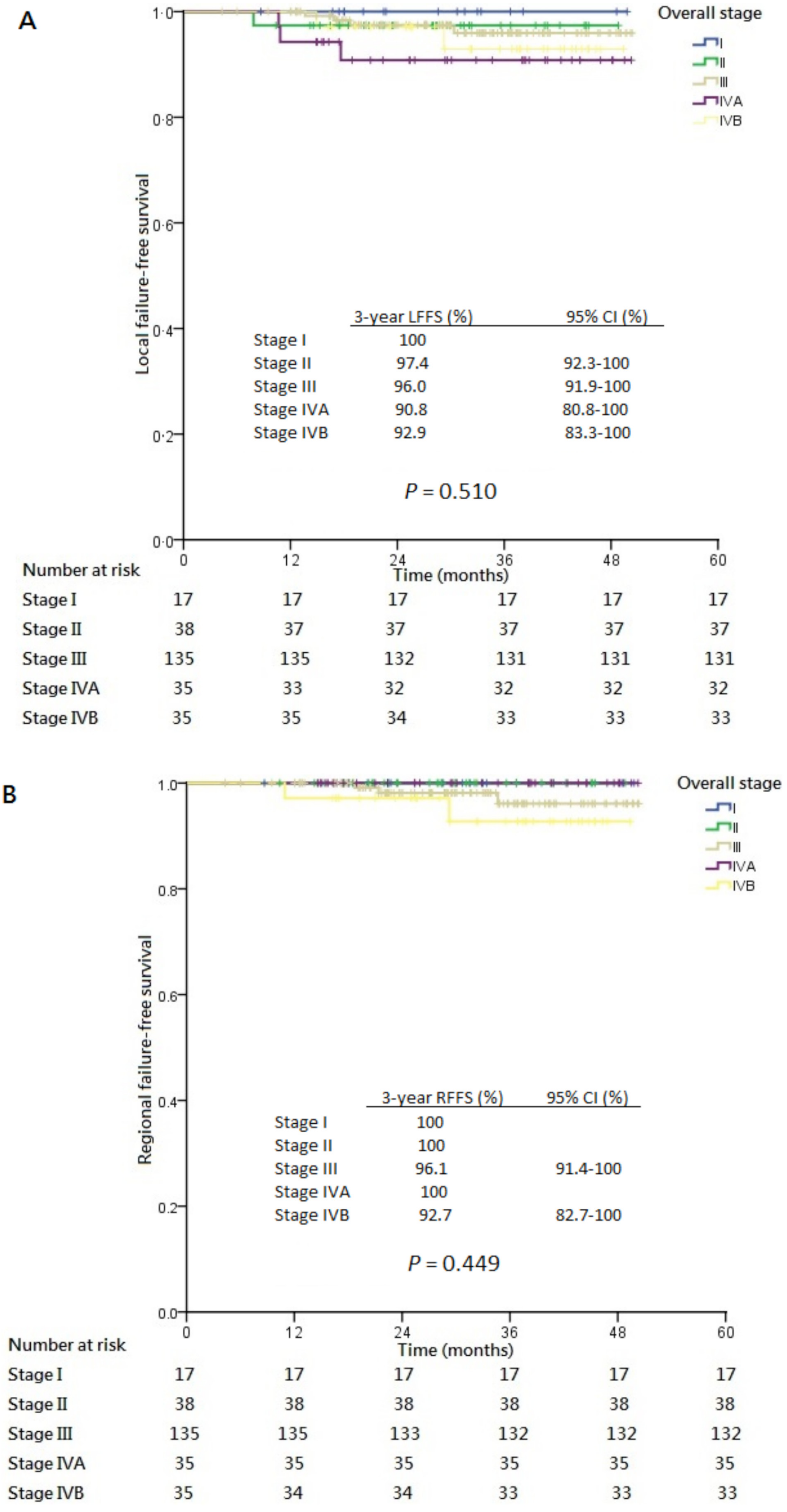

Supplementary Figure S1: Kaplan-Meier Estimates of All Survival Endpoints for All Stages of Nasopharyngeal Carcinoma. They indicate the data for panel **A.** local failure-free survival (LFFS), panel **B.** regional failure-free survival (RFFS).

(*Continued*)

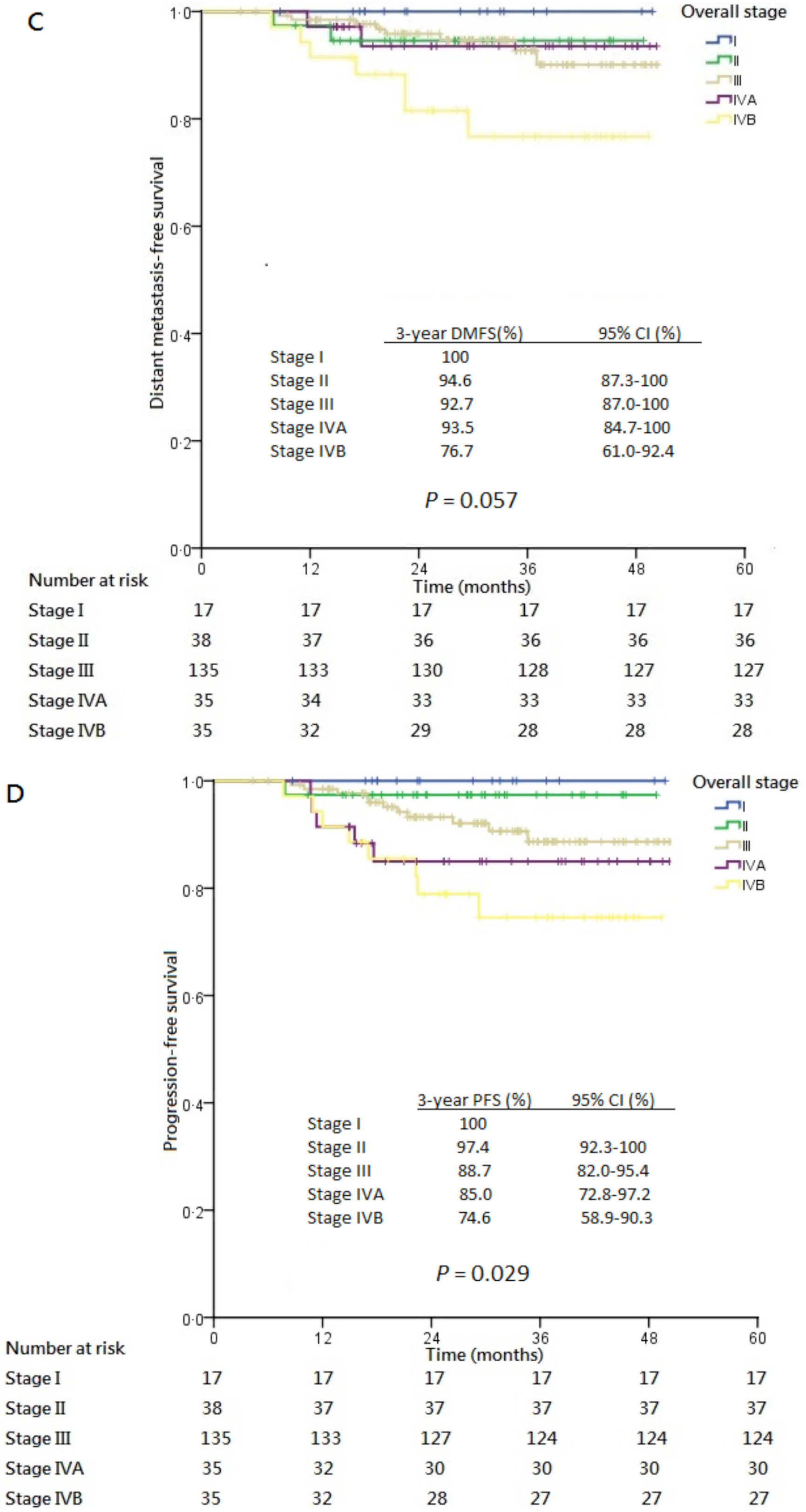

Supplementary Figure S1 (*Continued*): panel **C.** distant metastasis-free survival (DMFS), panel **D.** progression-free survival (PFS).

(*Continued*)

E

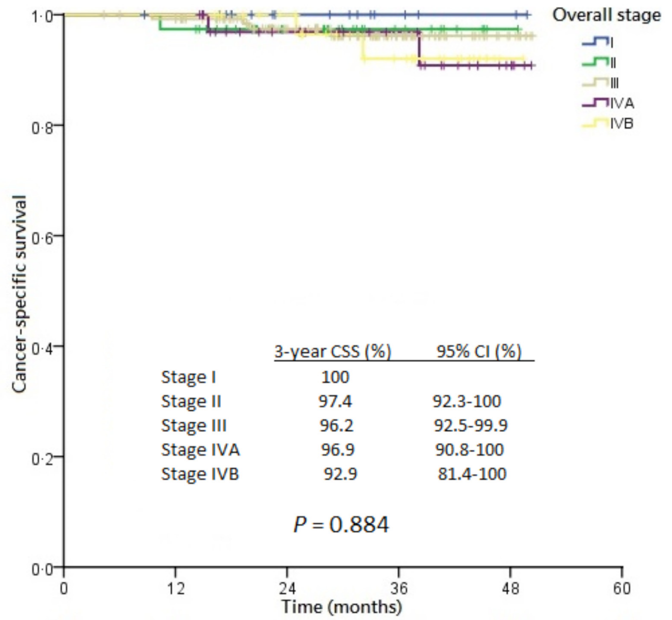

Number at risk						
Stage I	17	17	17	17	17	17
Stage II	38	37	37	37	37	37
Stage III	135	134	132	131	131	131
Stage IVA	35	35	34	34	33	33
Stage IVB	35	35	35	33	33	33

F

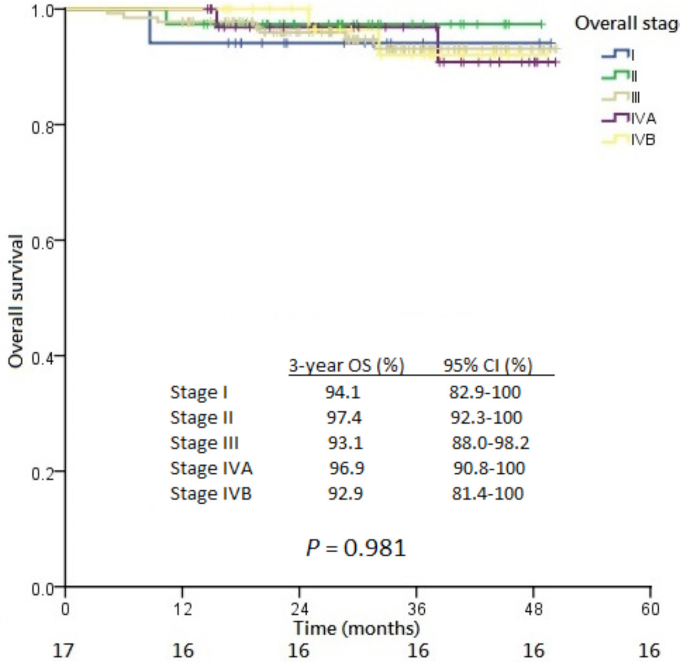

Number at risk						
Stage I	17	16	16	16	16	16
Stage II	38	37	37	37	37	37
Stage III	135	132	130	128	128	128
Stage IVA	35	35	34	34	33	33
Stage IVB	35	35	35	33	33	33

Supplementary Figure S1 (*Continued*): panel **E.** cancer-specific survival (CSS) and panel **F.** overall survival. CI: confidence interval.

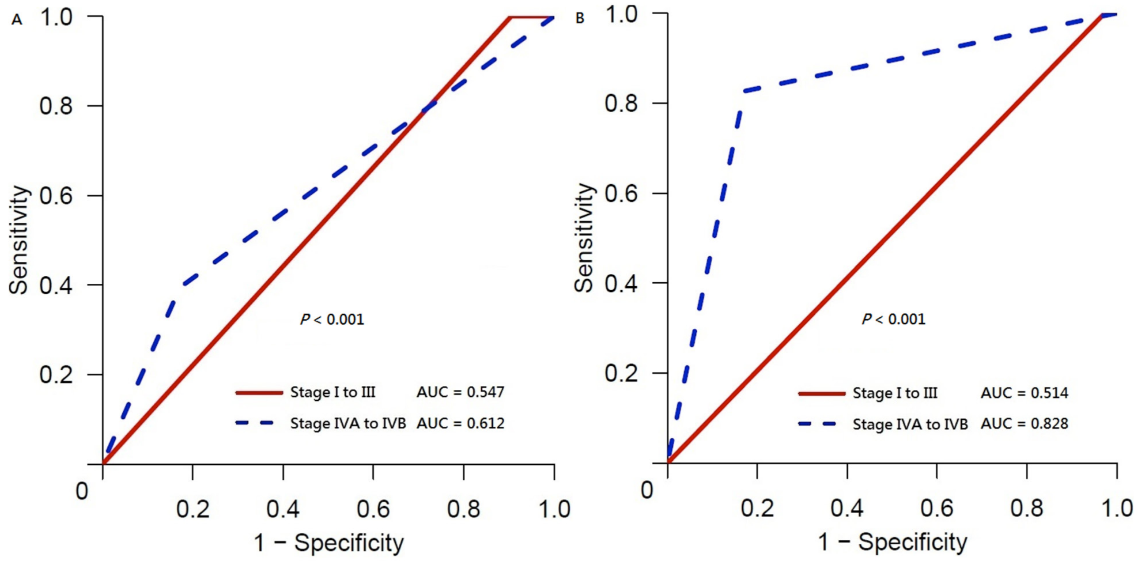

Supplementary Figure S2: Time-dependent receiver-operating characteristics (TDROC) curves of early post-IMRT undetectable plasma EBV DNA for stage I to III versus stage IVA to IVB nasopharyngeal carcinoma (NPC). The graphs indicate the data for panel **A** and **B.** local failure-free survival (LFFS) based on post-IMRT 8th week and 6th month undetectable plasma EBV DNA respectively.

(*Continued*)

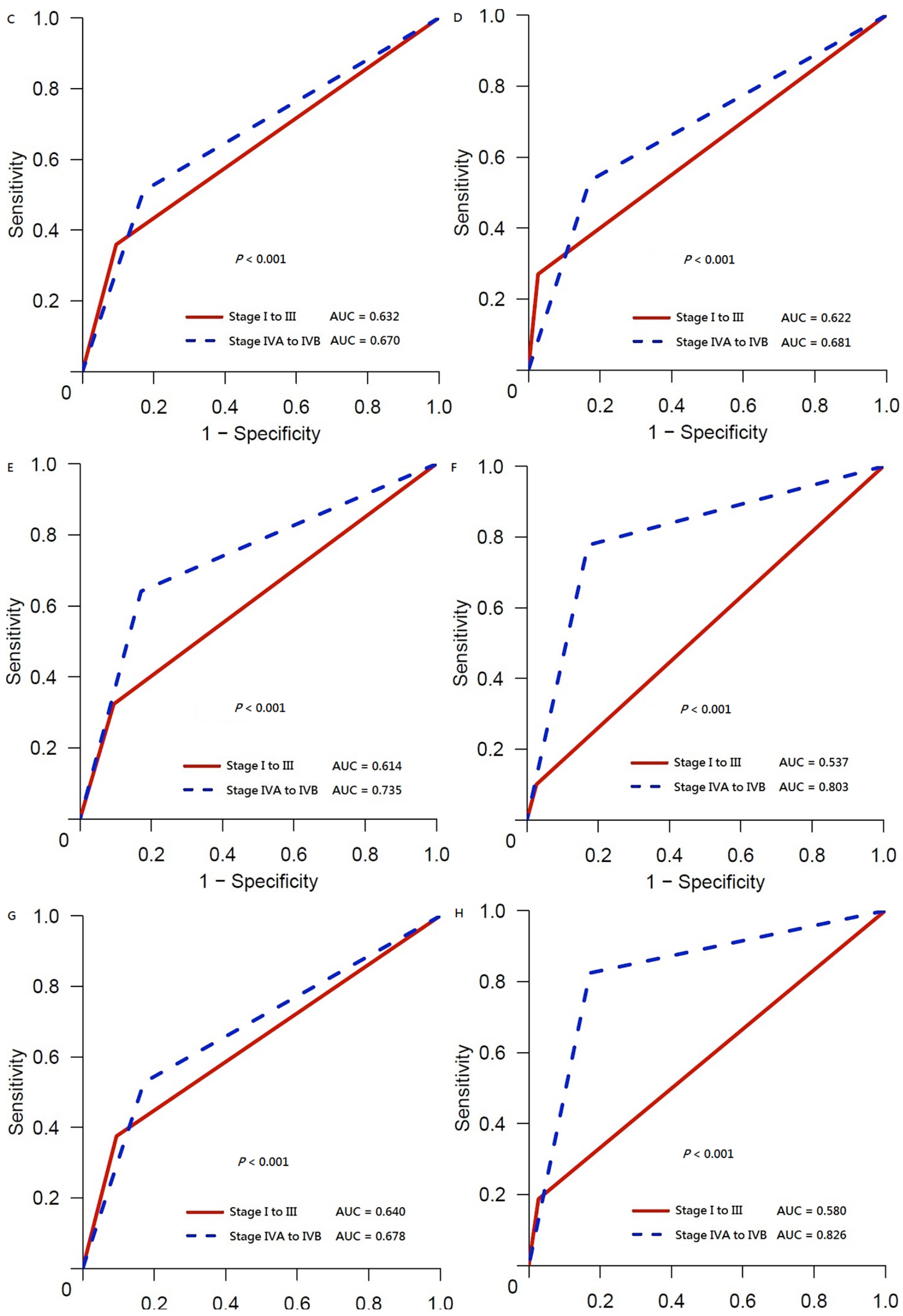

Supplementary Figure S2 (*Continued*): panel **C** and **D.** regional failure-free survival (RFFS) based on post-IMRT 8th week and 6th month undetectable plasma EBV DNA respectively; panel **E** and **F.** distant metastasis-free survival (DMFS) based on post-IMRT 8th week and 6th month undetectable plasma EBV DNA respectively; panel **G** and **H.** progression-free survival (PFS) based on post-IMRT 8th week and 6th month undetectable plasma EBV DNA respectively.

(Continued)

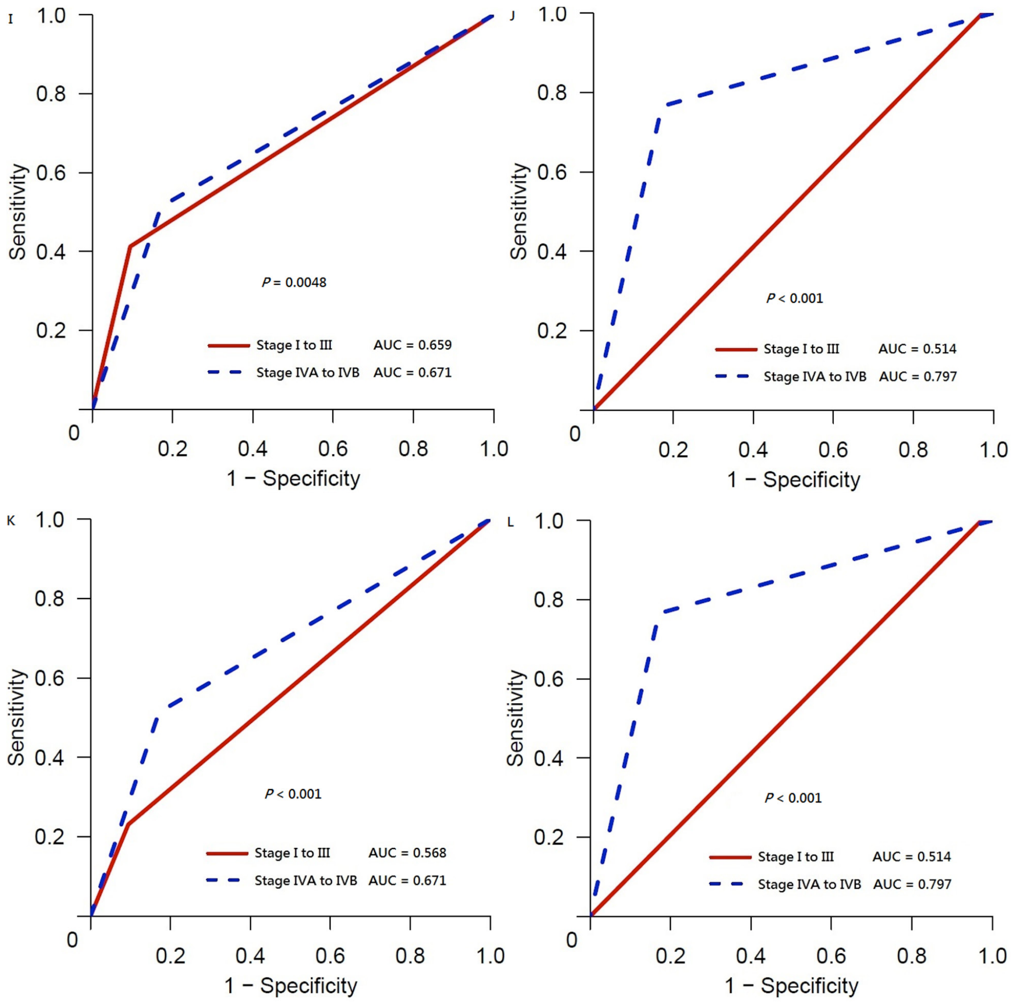

Supplementary Figure S2 (*Continued*): panel **I** and **J.** cancer-specific survival (CSS) based on post-IMRT 8th week and 6th month undetectable plasma EBV DNA respectively and panel **K** and **L.** overall survival (OS) based on post-IMRT 8th week and 6th month undetectable plasma EBV DNA respectively. IMRT: intensity-modulated radiation therapy.

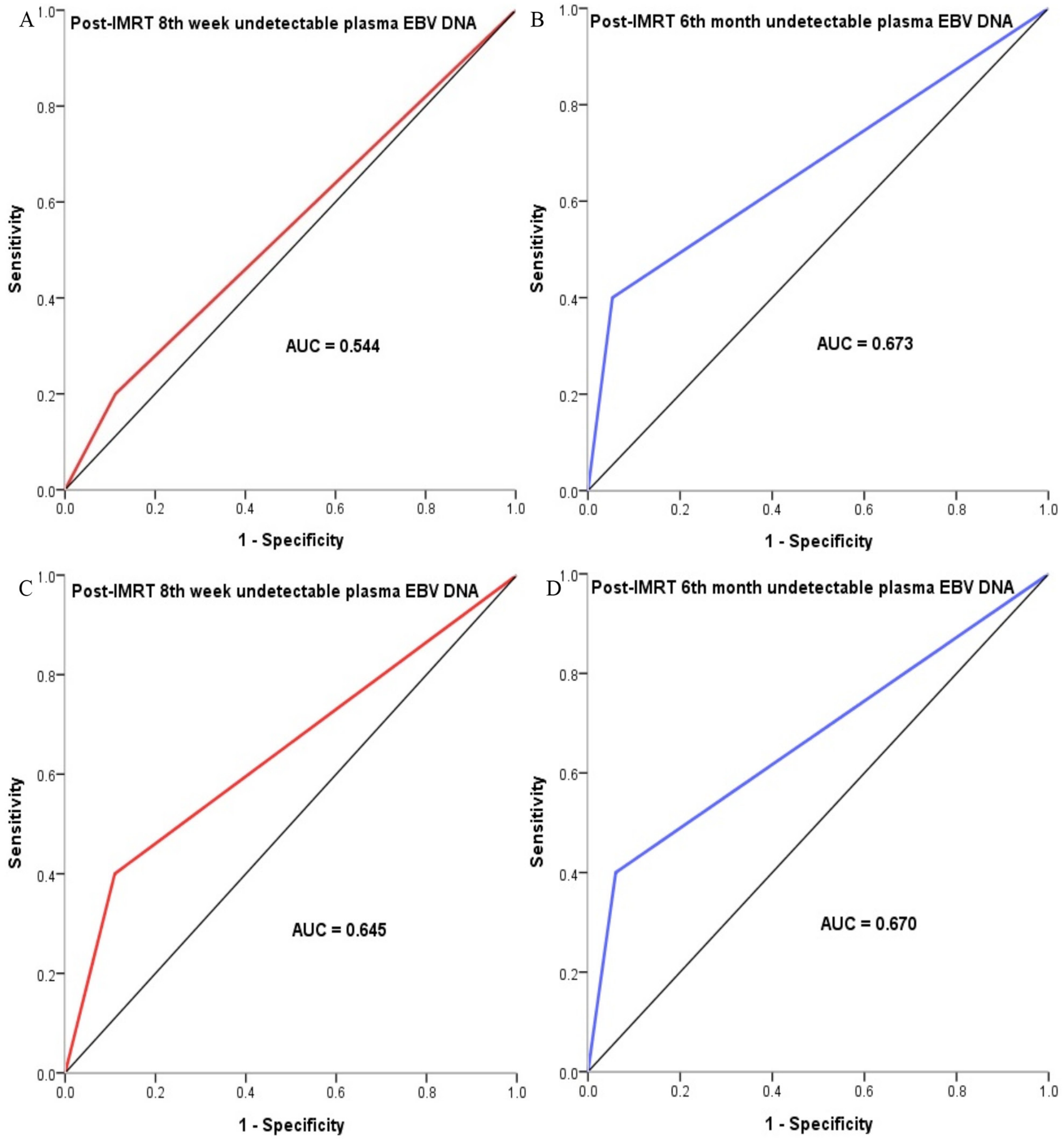

Supplementary Figure S3: Receiver-operating characteristics (ROC) curves for the performances of post-IMRT 8th week and post-IMRT 6th month undetectable plasma EBV DNA respectively. The graphs indicate the data for panel **A** and **B.** local failure-free survival, panel **C** and **D.** regional failure-free survival.

(*Continued*)

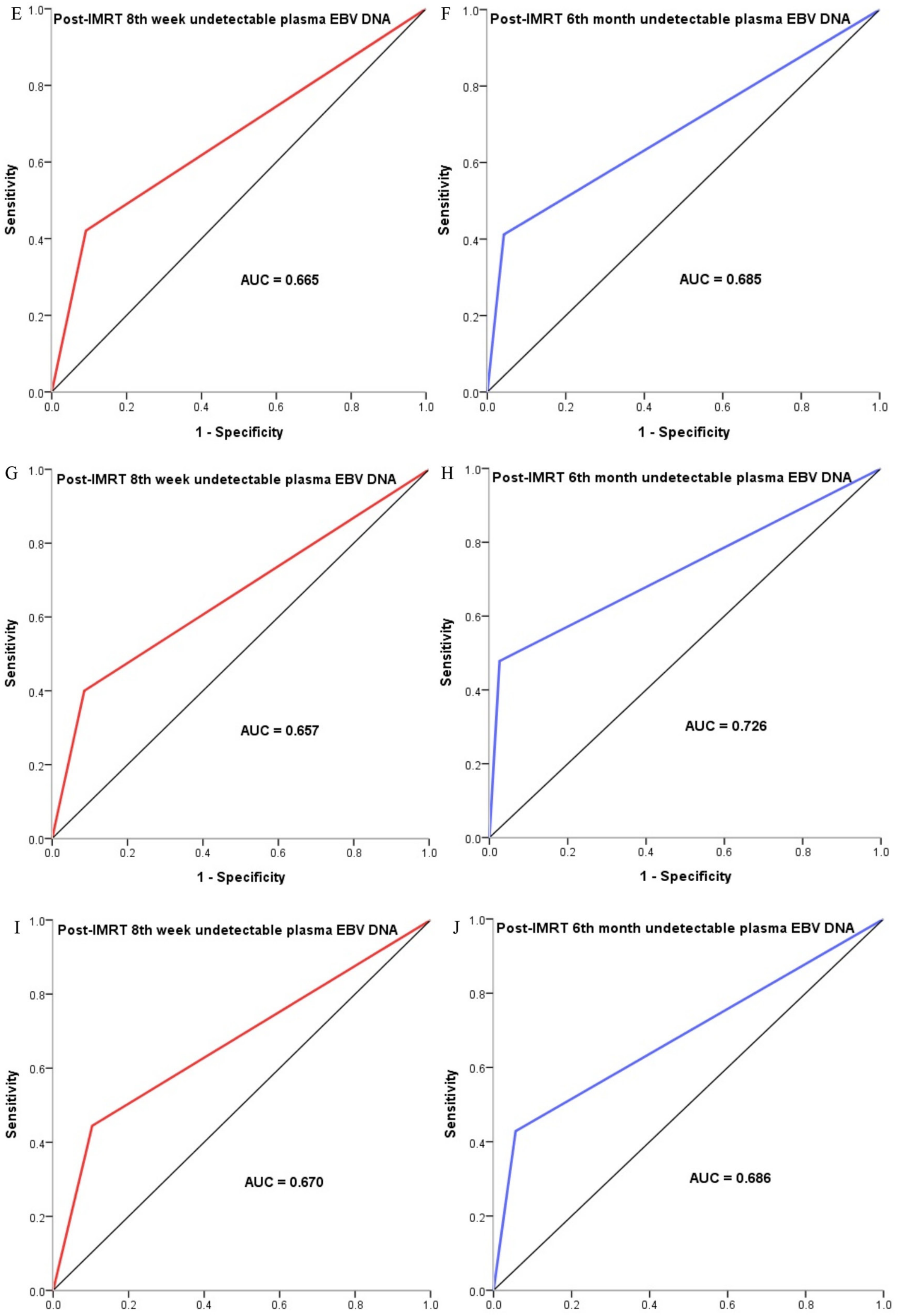

Supplementary Figure S3 (*Continued*): panel **E** and **F.** distant metastasis-free survival, panel **G** and **H.** progression-free survival, panel **I** and **J.** cancer-specific survival and,

(*Continued*)

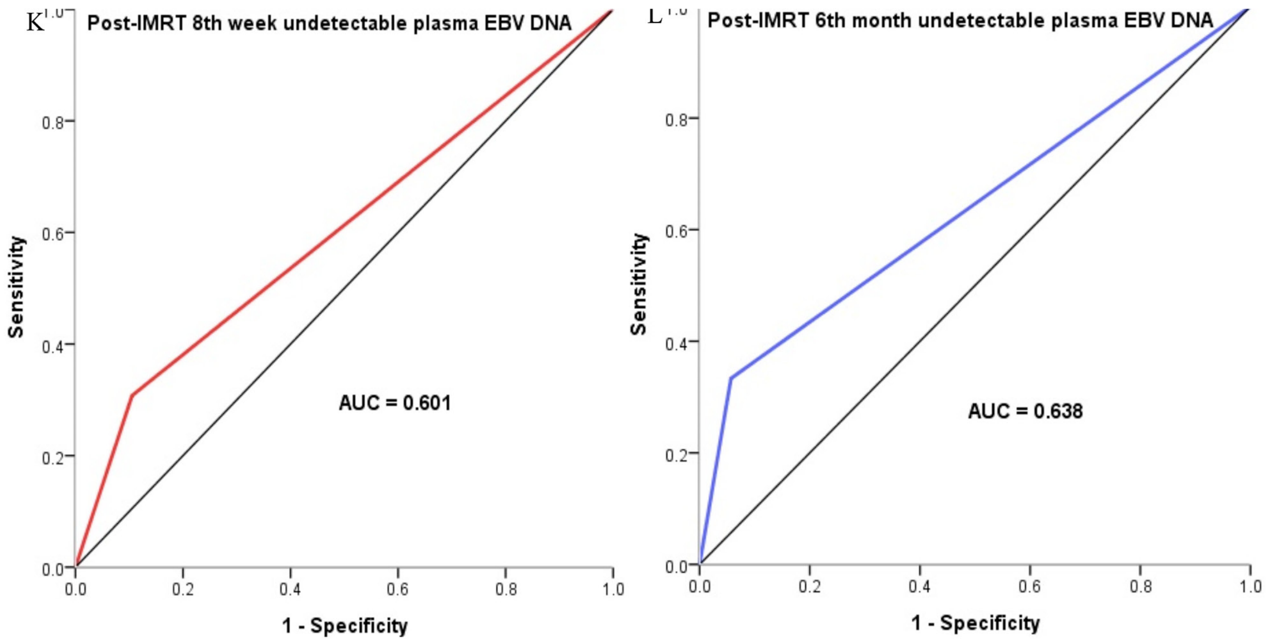

Supplementary Figure S3 (*Continued*): panel **K** and **L.** overall survival. AUC: area under the curve.

Supplementary Table S1: Univariable and multivariable Cox model for local failure-free survival and regional failure-free survival

See Supplementary File 1

Supplementary Table S2: Univariable and multivariable Cox model for distant metastasis-free survival and progression-free survival

See Supplementary File 2

Supplementary Table S3: Univariable and multivariable Cox model for cause-specific survival and overall survival

See Supplementary File 3

Supplementary Table S4: Performance indices of post-IMRT undetectable plasma EBV DNA on various survival endpoints

See Supplementary File 4
