# Supplementary material for: Prognostication of serial post-intensity-modulated radiation therapy undetectable plasma EBV DNA for nasopharyngeal carcinoma
Source: Oncotarget. 2016 Dec 24;8(3):5292–308. doi: 10.18632/oncotarget.14137 (PMC5354909; doi:10.18632/oncotarget.14137)
Supplement: Supplementary file 2 [file oncotarget-08-5292-s002.docx]

**Supplementary Table 1.** **Univariable and multivariable Cox model for local failure-free survival and regional failure-free survival**

|  | **Local failure-free survival** | | | | | | **Regional failure-free survival** | | | | | |
| --- | --- | --- | --- | --- | --- | --- | --- | --- | --- | --- | --- | --- |
|  | **Univariable analysis** | | | **Multivariable analysis**^*^ | | | **Univariable analysis** | | | **Multivariable analysis**^*^ | | |
|  | HR | 95% CI | *P* | HR | 95% CI | *P* | HR | 95% CI | *P* value | HR | 95% CI | *P* |
| Age | 0.99 | 0.95-1.04 | 0.667 | ND | | | 1.02 | 0.95-1.10 | 0.573 | ND | | |
| Sex (male) | 1.55 | 0.32-7.37 | 0.581 | ND | | | 1.26 | 0.56-3.31 | 0.972 | ND | | |
| ECOG PS | 0.05 | 0.02-3998.68 | 0.588 | ND | | | 3.76 | 0.42-33.62 | 0.238 | ND | | |
| T-classification | 0.47 | 0.56-1.20 | 0.223 | ND | | | 1.35 | 0.45-8.65 | 0.947 | ND | | |
| N-classification | 1.82 | 0.20-12.97 | 0.981 | ND | | | 0.48 | 0.03-7.68 | 0.599 | ND | | |
| Overall stage | 0.58 | 0.05-6.44 | 0.695 | ND | | | 0.38 | 0.06-2.25 | 0.887 | ND | | |
| IMRT alone vs chemoradiation | 0.38 | 0.28-9.99 | 0.372 | ND | | | 0.40 | 0.20-10.98 | 0.547 | ND | | |
| Concurrent chemoradiation only | 0.78 | 0.55-3.43 | 0.561 | ND | | | 0.67 | 0.57-5.86 | 0.902 | ND | | |
| Induction chemotherapy then concurrent chemoradiation | 0.55 | 0.16-1.91 | 0.346 | ND | | | 0.41 | 0.07-2.47 | 0.332 | ND | | |
| Concurent chemoradiation then adjuvant chemotherapy | 0.66 | 0.21-5.49 | 0.870 | ND | | | 0.59 | 0.11-8.87 | 0.964 | ND | | |
| Baseline plasma EBV DNA | 1.02 | 1.01-1.03 | 0.009 | 1.02 | 1.01-1.04 | 0.009 | 1.02 | 1.00-1.04 | 0.010 | 1.02 | 1.01-1.04 | 0.011 |
| Post-IMRT 8^th^ week undetectable plasma EBV DNA | 2.95 | 0.46-19.01 | 0.251 | ND | | | 0.18 | 0.03-1.08 | 0.061 | 0.14 | 0.02-0.93 | 0.041 |
| Post-IMRT 6^th^ month undetectable plasma EBV DNA | 0.05 | 0.01-0.24 | < .001 | 0.10 | 0.03-0.37 | .002 | 0.11 | 0.02-0.65 | 0.022 | 0.09 | 0.01-0.58 | 0.011 |

CI: confidence interval, ECOG: Eastern Cooperative Oncology Group, HR: hazard ratio, IMRT: intensity-modulated radiation therapy, ND: not done, PS: performance status.

^*^Only covariates found significant (*P* < 0.1) in the univariable analysis were considered in the multivariable analysis.
