# Supplementary material for: Prognostication of serial post-intensity-modulated radiation therapy undetectable plasma EBV DNA for nasopharyngeal carcinoma
Source: Oncotarget. 2016 Dec 24;8(3):5292–308. doi: 10.18632/oncotarget.14137 (PMC5354909; doi:10.18632/oncotarget.14137)
Supplement: Supplementary file 3 [file oncotarget-08-5292-s003.docx]

**Supplementary Table 2.** **Univariable and multivariable Cox model for distant metastasis-free survival and progression-free survival**

|  | **Distant metastasis-free survival** | | | | | | **Progression-free survival** | | | | | |
| --- | --- | --- | --- | --- | --- | --- | --- | --- | --- | --- | --- | --- |
|  | **Univariable analysis** | | | **Multivariable analysis**^*^ | | | **Univariable analysis** | | | **Multivariable analysis**^*^ | | |
|  | HR | 95% CI | *P* | HR | 95% CI | *P* | HR | 95% CI | *P* | HR | 95% CI | *P* |
| Age | 0.99 | 0.96–1.02 | 0.601 | ND | | | 1.00 | 0.97–1.03 | 0.870 | ND | | |
| Sex (male) | 1.21 | 0.43–3.40 | 0.719 | ND | | | 1.30 | 0.52–3.29 | 0.575 | ND | | |
| ECOG PS | 3.21 | 0.93–11.01 | 0.137 | ND | | | 2.29 | 0.68–7.64 | 0.179 | ND | | |
| T-classification | 2.01 | 0.42–9.70 | 0.394 | ND | | | 0.62 | 0.30–1.84 | 0.386 | ND | | |
| N-classification | 0.18 | 0.02–1.45 | 0.116 | ND | | | 0.29 | 0.06–1.40 | 0.122 | ND | | |
| Overall stage | 0.28 | 0.06–1.34 | 0.165 | ND | | | 0.62 | 0.20–1.91 | 0.120 | ND | | |
| IMRT alone vs chemoradiation | 0.29 | 0.04–2.18 | 0.227 | ND | | | 0.44 | 0.10–1.85 | 0.258 | ND | | |
| Concurrent chemoradiation only | 0.76 | 0.67–1.68 | 0.970 | ND | | | 0.91 | 0.79–1.58 | 0.951 | ND | | |
| Induction chemotherapy then concurrent chemoradiation | 0.55 | 0.46–1.36 | 0.252 | ND | | | 0.59 | 0.27–1.28 | 0.182 | ND | | |
| Concurrent chemoradiation then adjuvant chemotherapy | 0.63 | 0.56–1.32 | 0.313 | ND | | | 0.68 | 0.53–1.46 | 0.251 | ND | | |
| Baseline plasma EBV DNA | 1.02 | 1.01–1.03 | < 0.001 | 1.02 | 1.01–1.04 | 0.004 | 1.02 | 1.01–1.03 | < 0.001 | 1.02 | 1.01–1.03 | 0.002 |
| Post-IMRT 8^th^ week undetectable plasma EBV DNA | 0.14 | 0.06–0.36 | < 0.001 | 0.12 | 0.05–0.31 | < 0.001 | 0.15 | 0.07–0.35 | < 0.001 | 0.14 | 0.06–0.31 | < 0.001 |
| Post-IMRT 6^th^ month undetectable plasma EBV DNA | 0.09 | 0.02–0.33 | < 0.001 | 0.06 | 0.02–0.17 | < 0.001 | 0.05 | 0.02–0.11 | < 0.001 | 0.04 | 0.02–0.10 | < 0.001 |

CI: confidence interval, ECOG: Eastern Cooperative Oncology Group, HR: hazard ratio, IMRT: intensity-modulated radiation therapy, ND: not done, PS: performance status.

^*^Only covariates found significant (*P* < 0.1) in the univariable analysis were considered in the multivariable analysis.
