# Supplementary material for: Prognostication of serial post-intensity-modulated radiation therapy undetectable plasma EBV DNA for nasopharyngeal carcinoma
Source: Oncotarget. 2016 Dec 24;8(3):5292–308. doi: 10.18632/oncotarget.14137 (PMC5354909; doi:10.18632/oncotarget.14137)
Supplement: Supplementary file 4 [file oncotarget-08-5292-s004.docx]

**Supplementary Table 3. Univariable and multivariable Cox model for cause-specific survival and overall survival**

|  | **Cause-specific survival** | | | | | | **Overall survival** | | | | | |
| --- | --- | --- | --- | --- | --- | --- | --- | --- | --- | --- | --- | --- |
|  | **Univariable analysis** | | | **Multivariable analysis**^*^ | | | **Univariable analysis** | | | **Multivariable analysis**^*^ | | |
|  | HR | 95% CI | *P* | HR | 95% CI | *P* | HR | 95% CI | *P* | HR | 95% CI | *P* |
| Age | 1.05 | 1.00–1.11 | 0.071 | 1.08 | 1.02–1.10 | 0.094 | 1.05 | 1.00–1.09 | 0.032 | 1.06 | 1.00–1.12 | 0.041 |
| Sex (male) | 0.85 | 0.20–3.63 | 0.825 | ND | | | 0.47 | 0.15–1.46 | 0.192 | ND | | |
| ECOG PS | 4.45 | 0.92–21.50 | 0.148 | ND | | | 2.82 | 0.63–12.74 | 0.180 | ND | | |
| T-classification | 0.63 | 0.09–4.55 | 0.646 | ND | | | 0.92 | 0.15–5.55 | 0.928 | ND | | |
| N-classification | 1.26 | 0.18–8.96 | 0.824 | ND | | | 1.82 | 0.30–10.93 | 0.514 | ND | | |
| Overall stage | 1.00 | 0.14–7.35 | 0.931 | ND | | | 1.14 | 0.16–8.37 | 0.847 | ND | | |
| IMRT alone vs chemoradiation | 2.92 | 0.73–11.73 | 0.130 | ND | | | 0.19 | 0.02–1.63 | 0.128 | ND | | |
| Concurrent chemoradiation only | 0.97 | 0.75–1.30 | 0.983 | ND | | | 0.98 | 0.76–1.26 | 0.990 | ND | | |
| Induction chemotherapy then concurrent chemoradiation | 1.28 | 0.32–5.14 | 0.729 | ND | | | 2.68 | 0.73–9.92 | 0.142 | ND | | |
| Concurent chemoradiation then adjuvant chemotherapy | 0.85 | 0.66–2.36 | 0.357 | ND | | | 0.88 | 0.57–2.10 | 0.287 | ND | | |
| Baseline plasma EBV DNA | 0.99 | 0.88–1.11 | 0.820 | ND | | | 0.97 | 0.76–1.23 | 0.786 | ND | | |
| Post-IMRT 8^th^ week undetectable plasma EBV DNA | 0.16 | 0.04–0.60 | 0.009 | 0.18 | 0.06–0.67 | 0.031 | 0.29 | 0.09–0.94 | 0.041 | 0.31 | 0.10–1.00 | 0.049 |
| Post-IMRT 6^th^ month undetectable plasma EBV DNA | 0.11 | 0.03–0.49 | 0.004 | 0.12 | 0.02–0.47 | 0.004 | 0.17 | 0.04–0.67 | 0.012 | 0.16 | 0.02–0.71 | 0.019 |

CI: confidence interval, ECOG: Eastern Cooperative Oncology Group, HR: hazard ratio, IMRT: intensity-modulated radiation therapy, ND: not done, PS: performance status.

^*^Only covariates found significant (*P* < 0.1) in the univariable analysis were considered in the multivariable analysis.
