# Supplementary material for: Prognostication of serial post-intensity-modulated radiation therapy undetectable plasma EBV DNA for nasopharyngeal carcinoma
Source: Oncotarget. 2016 Dec 24;8(3):5292–308. doi: 10.18632/oncotarget.14137 (PMC5354909; doi:10.18632/oncotarget.14137)
Supplement: Supplementary file 5 [file oncotarget-08-5292-s005.docx]

**Supplementary Table 4. Performance indices of post-IMRT undetectable plasma EBV DNA on various survival endpoints**

|  | **Post-IMRT 8^th^ week undetectable**  **plasma EBV DNA** | | | **Post-IMRT 6^th^ month undetectable plasma EBV DNA** | | |
| --- | --- | --- | --- | --- | --- | --- |
|  | AUC | Cτ | 95% CI | AUC | Cτ | 95% CI |
| **LFFS** |  | | | | | |
| stage I–III | 0.547 | 0.547 | 0.52–0.57 | 0.514 | 0.516 | 0.50–0.53 |
| stage IVA–B | 0.612 | 0.641 | 0.45–0.83 | 0.828 | 0.858 | 0.71–1.00 |
| *P* | < 0.001 | | | < 0.001 | | |
| **RFFS** |  | | | | | |
| stage I–III | 0.632 | 0.675 | 0.41–0.94 | 0.622 | 0.678 | 0.38–0.98 |
| stage IVA–B | 0.670 | 0.744 | 0.48–1.01 | 0.681 | 0.741 | 0.47–1.01 |
| *P* | < 0.001 | | | < 0.001 | | |
| **DMFS** |  | | | | | |
| stage I–III | 0.614 | 0.644 | 0.48-0.81 | 0.537 | 0.575 | 0.45–0.71 |
| stage IVA–B | 0.735 | 0.741 | 0.58-0.90 | 0.803 | 0.814 | 0.68–0.95 |
| *P* | < 0.001 | | | < 0.001 | | |
| **PFS** |  | | | | | |
| stage I–III | 0.640 | 0.651 | 0.50–0.80 | 0.580 | 0.601 | 0.46–0.74 |
| stage IVA–B | 0.678 | 0.670 | 0.53–0.81 | 0.826 | 0.826 | 0.72–0.94 |
| *P* | < 0.001 | | | < 0.001 | | |
| **CSS** |  | | | | | |
| stage I–III | 0.659 | 0.679 | 0.47–0.89 | 0.514 | 0.517 | 0.50–0.53 |
| stage IVA–B | 0.671 | 0.694 | 0.45–0.94 | 0.797 | 0.771 | 0.50–1.04 |
| *P* | 0.0048 | | | < 0.001 | | |
| **OS** |  | | | | | |
| stage I–III | 0.568 | 0.584 | 0.46–0.71 | 0.514 | 0.517 | 0.50–0.53 |
| stage IVA–B | 0.671 | 0.694 | 0.45–0.94 | 0.797 | 0.771 | 0.50–1.04 |
| *P* | < 0.001 | | | < 0.001 | | |

AUC: area under the curve, CI: confidence interval, CSS: cancer-specific survival, Cτ: time-dependent concordance index, DMFS: distant metastasis-free survival, IMRT: intensity-modulated radiation therapy, LFFS: local failure-free survival, OS: overall survival, PFS: progression-free survival, RFFS: regional failure-free survival.
